# The Many Hidden Faces of Gallbladder Carcinoma on CT and MRI Imaging—From A to Z

**DOI:** 10.3390/diagnostics14050475

**Published:** 2024-02-22

**Authors:** Damaris Neculoiu, Lavinia Claudia Neculoiu, Ramona Mihaela Popa, Rosana Mihaela Manea

**Affiliations:** 1 Department of Radiology and Medical Imaging, Clinical Emergency County Hospital of Brașov, 500326 Brașov, Romania; 2 Faculty of Medicine, “Transilvania” University of Brașov, Nicolae Bălcescu 56, 500019 Brașov, Romania

**Keywords:** gallbladder, gallbladder cancer, biliary tract cancer, CT, MRI, oncology, surgery, metastases

## Abstract

Gallbladder carcinoma represents the most aggressive biliary tract cancer and the sixth most common gastrointestinal malignancy. The diagnosis is a challenging clinical task due to its clinical presentation, which is often non-specific, mimicking a heterogeneous group of diseases, as well as benign processes such as complicated cholecystitis, xanthogranulomatous cholecystitis, adenomyomatosis, porcelain gallbladder or metastasis to the gallbladder (most frequently derived from melanoma, renal cell carcinoma). Risk factors include gallstones, carcinogen exposure, porcelain gallbladder, typhoid carrier state, gallbladder polyps and abnormal pancreaticobiliary ductal junction. Typical imaging features on CT or MRI reveal three major patterns: asymmetric focal or diffuse wall-thickening of the gallbladder, a solid mass that replaces the gallbladder and invades the adjacent organs or as an intraluminal enhancement mass arising predominantly from the gallbladder fundus. The tumor can spread to the liver, the adjacent internal organs and lymph nodes. Depending on the disease stage, surgical resection is the curative treatment option in early stages and adjuvant combination chemotherapy at advanced stages. The purpose of this scientific paper is to fully illustrate and evaluate, through multimodality imaging findings (CT and MRI), different presentations and imaging scenarios of gallbladder cancer in six patients and thoroughly analyze the risk factors, patterns of spread and differential diagnosis regarding each particular case.

Gallbladder cancer is the most common malignancy of the biliary tract and the sixth most common cancer of the gastrointestinal system [1]. According to GLOBOCAN estimates, gallbladder cancer is relatively rare and stands in 24th place among the most frequent type of cancers worldwide with more than 115,949 new cases in 2020 [2,3]. In the majority of cases, gallbladder carcinoma is asymptomatic or the clinical presentation is often vague, non-specific and discovered at an advanced stage [4,5]. Imaging plays a crucial and decisive role in the diagnosis, staging and subsequent management planning [6]. Occasionally, gallbladder cancer might be discovered following a cholecystectomy. Moreover, gallbladder cancer is thought to be favored by chronic cholelithiasis, cholecystolithiasis, gallbladder polyps and porcelain gallbladder [7]. The prevalence of the disease is primarily among elderly women over 60 years-old. The highest incidence occurs in South American countries, Chile, Ecuador, India, Pakistan, Japan and South Korea. Incidence of gallbladder cancer is 1–2 cases per 100,000 people [3,8,9]. However, gallbladder carcinoma still remains a relatively rare pathology with a poor prognosis and it usually presents at a very advanced stage [1]. Late-stage illness frequently manifests with anorexia, weight loss, abdominal pain and jaundice [3].

Diagnostic imaging modalities for the gallbladder cancer include ultrasound, computerized tomography (CT) and magnetic resonance imaging (MRI). CT and MRI are both effective imaging modalities, but MRI provides superior soft-tissue characterization of the gallbladder and biliary tree. The use of hepatobiliary contrast agents (gadolinium chelates) with increased hepatobiliary excretion in abdominal MRI imaging may offer valuable information by providing enhanced images of the biliary tree [10].

We hereby fully illustrate the case of a 67-year-old female patient, who was admitted to the Emergency Department with intense pain localized in the right renal fossa, radiating to the right abdominal flank, accompanied by nausea with an onset of approximately two weeks. During the physical examination, a reduced abdominal wall mobility with respiratory movements was observed, along with pain in the right hypochondrium and muscular defense. Her medical history included hypertension grade 3 and congestive heart failure. Laboratory tests showed elevated inflammatory markers (leukocytosis, procalcitonin, CRP) and hypochromic microcytic anemia.

Biphasic (arterial phase followed by venous phase) contrast-enhanced emergency CT was performed (Figure 1), which clearly highlighted a gallbladder hydrops, with asymmetric, irregular gallbladder mural thickening, associated with multiple intraluminal mixed stones (Panel A). The tumoral mass extends directly into the adjacent liver parenchyma in segments IV and V and is in contact with the ascending colon (Panel C). Hepatomegaly can be observed (right hepatic lobe measures = 190 mm), with nodular lesions, disseminated in both hepatic lobes, hypodense, with rim peripheral contrast enhancement, more numerous in the right lobe, presenting various sizes (up to 35 mm in segment V) highly suggestive of liver metastases (Panel B and Panel C). Lymphatic metastases are common in gallbladder cancer. In this particular case, CT showed multiple lymph nodes enlargements in the hilar, mesenteric, celiac and precaval regions, up to 26/25 mm (measured in the hepatic hilum), with associated central necrosis (Panel D).

The patient was transferred to the General Surgery Department for specialized treatment (intravenous antibiotics, intravenous hydration and correction of electrolyte abnormalities). After laparoscopy and laparotomy, a subhepatic perforated tumor with duodenum and transvers colon invasion was revealed. A partial cholecystectomy was performed with cholecystostomy and intraperitoneal drain. The postoperative evolution progressed without incident.

Formalin-fixed paraffin-embedded tissue sections from gallbladder and liver were examined histologically. The microscopic description was suggestive of poorly differentiated gallbladder adenocarcinoma (G3); pT3NxMx. The liver metastatic site was pathologically confirmed. TNM according to the AJCC (American Joint Committee on Cancer) 8^th^ edition gallbladder cancer staging system was in this case T3N2M1. Oncology follow-up and adjuvant chemotherapy were recommended.

A 55-year-old female patient was admitted to the Emergency Department with right hypochondrium pain and weight loss for 2 weeks, which had worsened over the last two days accompanied by nausea and vomiting. No medical history was noted. Physical examination revealed normal abdominal wall mobility with respiratory movements and a sensitive right hypochondrium. Blood sample demonstrated normal levels of leukocytes and inflammatory markers.

Contrast-enhanced emergency CT (Figure 2) revealed mucosal hyperenhancement of the gallbladder, with irregular, mural thickening (16 mm), a gallstone (15 mm) and pericholecystic fluid and loco-regional inflammatory reactive lymph nodes (Panel A, Panel B and Panel C).

The CT scan depicted an abscess adjacent to the gallbladder with subtle peripheral contrast enhancement, measuring up to 12 mm in size along with inflammatory alterations in the adjacent hepatic parenchyma (Panel D). Inflammatory fat stranding can be observed at the omentum, periduodenum and pericolonic areas, as well as free intraperitoneal fluid.

An acute cholecystitis complicated by pericholecystic abscess was diagnosed. The patient refused hospitalization and specialized treatment. The following day the patient returned to the Emergency Department with severe pain and was admitted directly to the General Surgery Department. Nevertheless, blood sample demonstrated increased levels of CEA (67.83 ng/mL) and CA 19-9 (110.20 U/mL), markers which brought to question the CT imaging diagnosis of an acute cholecystitis complicated by pericholecystic abscess.

Therefore, clinical suspicion of gallbladder carcinoma was raised and an MRI cholangiography was performed (Figure 3). A laparoscopic cholecystectomy was performed and a subhepatic tumoral block with transvers colon invasion was identified. The patient was referred to the Oncology Department for further specialized treatment and follow-up.

A 49-year-old woman with no relevant medical history presented with a 1-week his-tory of abdominal pain, jaundice, dark-colored urine and clay-colored stool. Physical examinations revealed normal abdominal wall mobility with respiratory movements, pain and abdominal tenderness in the epigastric region. Laboratory results upon admission revealed elevated transaminases (ASAT 314 U/L, ALAT 484 U/L) and icteric cholestasis (GGT 311 U/L, bilirubin 8.59 mg/dL). The complete blood count was normal.

Contrast-enhanced emergency CT was performed (Figure 4), which richly highlighted a large heterogeneous intraluminal gallbladder mass, localized in the gallbladder fundus, measuring 58/34 mm, irregular, peripheral contrast enhancement on arterial and venous phase and with central hypodensity suggestive of areas of necrosis, extending to the surrounding liver (segment V) (Panel A and Panel C). CT showed a gallstone (17 mm) wedged in the gallbladder neck and intrahepatic biliary dilatation (Panel C and D). The common bile duct was dilated due to the presence of a possible tumoral extension to biliary tract or by the compressive effect of the multiple hilar lymphadenopathies; mesenteric, celiac and retroperitoneal lymphadenopathies with areas of necrosis, measuring up to 25/15 mm were also noted (Panel B). Abdominal contrast-enhanced MRI was performed (Figure 5).

Furthermore, endoscopic retrograde cholangiopancreatography showed a stenosis (with the length of 14–15 mm) at the middle third of the major biliary tract, therefore a stent was placed.

In this case, the probable diagnosis was of synchronous gallbladder and biliary tract carcinoma with multiple large lymphadenopathies localized in the hepatic hilum, surrounding the cephalic region of the pancreas and in the celiac region.

Endoscopic ultrasound-guided fine-needle aspiration (EUS-FNA) for gallbladder tissue was performed. Histopathology showed small cell neuroendocrine carcinoma of the gallbladder. Immunohistochemical stains were positive for CK7, synaptophysin (Syn) and chromogranin A (CgA), and the Ki-67 indexes were over 97% cells.

A 69-year-old female patient with a past medical history of diabetes type II presented to the Emergency Department with a 2-day upper abdominal pain, accompanied by hypotension and oligoanuria. Routine laboratory evaluation showed elevated inflammatory markers (leukocytosis, procalcitonin 100 ng/mL, CRP 126 mg/L), elevated transaminases and ferritin. The patient underwent contrast-enhanced computed tomography (Figure 6). CT images depicted a distended gallbladder (99 mm in longitudinal measurement), with asymmetrical thick-walled gallbladder (16 mm), heterogeneous contrast enhancement (Panel A), with a gallbladder neck stone (10 mm), extended to the duodenum (Panel B and Panel C). CT showed multiple low-attenuation hepatic masses with peripheral enhancement, adjacent to the gallbladder fossa (segment V) and intrahepatic biliary tract dilatation. Below the liver and adjacent to the gallbladder fundus, fat standing and free fluid were observed. These imaging findings were suggestive of acute cholecystitis complicated by an intrahepatic abscess or gallbladder carcinoma with wall perforation into the adjacent liver.

Antibiotic therapy and percutaneous US-guided drainage for liver abscess represented the first-line treatment, without response. After that, surgical drainage and cholecystostomy was performed.

Histopathological analysis revealed gallbladder carcinoma and palliative chemotherapy was proposed.

A 63-year-old man with a history of severe hyponatremia, known prostate adenocarcinoma and gastroduodenal ulcer with Billroth I gastric resection presented with nausea, vomiting, dizziness and weight loss for one month. On physical examination, abdominal tenderness was noted. Routine laboratory evaluation demonstrated normal leukocytes and inflammatory markers, moderate anemia and severe hyponatremia (serum sodium was 108 mmol/L). Contrast-enhanced CT (Figure 7) showed a heterogeneous intraluminal gallbladder mass, measuring 25/24/35 mm, localized in gallbladder fundus (Panel A, Panel B, Panel C and Panel D). The mass had no invasion of the adjacent structures and no associated imaging findings. An abdominal MRI was performed (Figure 8).

Furthermore, a laparoscopic cholecystectomy was performed. The histopathological exam revealed gallbladder carcinoma.

The particularity of this case report is amply illustrated by severe hyponatremia presented as paraneoplastic SIADH syndrome (syndrome of inappropriate antidiuretic hormone secretion) in a patient with gallbladder carcinoma.

A 67-year-old female patient with a past medical history of hypertension and autoimmune thyroiditis presented to the Emergency Department with abdominal pain accompanied by nausea and weight loss. Physical examinations revealed abdominal tenderness with a palpable mass in the right hypochondrium. Blood sample demonstrated elevated inflammatory markers, hypochromic microcytic anemia, hepatic cytolysis and increased levels of CEA (12.5 ng/mL) and CA 19-9 (51 U/mL). Contrast-enhanced emergency CT (Figure 9) showed a large mass with heterogeneous enhancement, measuring 94/57 mm, that partially replaced the gallbladder and invaded the liver (segment IVb), pyloric antrum and duodenum II. Bulky celiac and mesenteric lymphadenopathies with areas of necrosis, measuring up to 27/25 mm were present.

Abdominal MRI was performed (Figure 10).

Endoscopic ultrasound-guided fine-needle aspiration (EUS-FNA) for gallbladder tissue was performed and revealed epithelial gallbladder carcinoma. The biopsy specimens were processed for frozen sectioning. Formalin-fixed paraffin-embedded tissue sections from the gallbladder were examined histologically. The microscopic description revealed proliferation of polygonal cells, abundant clear cytoplasm, large nuclei with irregular membranes and atypical mitotic divisions.

The clinical symptoms of gallbladder cancer are often vague and non-specific and include pain in the right hypochondriac region, nausea and vomiting. In the late stages of the disease weight loss, anorexia and jaundice are often seen [11,12]. In contrast, some patients present with symptoms of acute cholecystitis and malignancy may be incidentally found following a cholecystectomy [13]. Detection of gallbladder at an early stage is difficult because the symptoms often mimic benign conditions.

The major risk factors include being an elderly woman (over 60 years old, F:M ratio 3:1), cholelithiasis and gallstones (in 60–90% cases) [10]. Regarding our cases, five of them were female and one was male. Other risk factors include:

✓ chronic inflammation due to typhoid carrier state;
✓ gallbladder polyps (more than 10 mm);
✓ porcelain gallbladder;
✓ smoking and obesity [3,11];
✓ anomalous pancreaticobiliary ductal junction, which is a rare congenital anomaly [12].

Gallbladder carcinoma might be found incidentally in 1–3% following a cholecystectomy [14]. Because of advanced disease at diagnosis, the typical 5-year survival is only 5% [15].

Imaging has a key role in the diagnosis, staging, characterization and planning management of gallbladder cancer.

Diagnostic imaging modalities for the gallbladder cancer include ultrasound, computerized tomography (CT) and magnetic resonance imaging (MRI). Ultrasound is frequently the initial imaging modality for evaluating gallbladder disease. In locally advanced gallbladder cancer, ultrasound has a sensitivity of 85% and a specificity of 80% in diagnosis. Moreover, ultrasound is limited to evaluate locoregional extension, nodal and metastatic disease. CT and MRI are commonly indicated for the comprehensive assessment of disease extension. Biphasic arterial phase (at 20 to 30 s) followed by venous phase (50 to 60 s) contrast-enhanced CT is useful to evaluate gallbladder cancer. CT demonstrates a sensitivity of 99% and a specificity of 76% in determining resectability. MRI is a noninvasive imaging method and demonstrates superior sensitivity compared to CT, providing superior soft-tissue characterization of the gallbladder and biliary tree [10].

Computed tomography (CT) and magnetic resonance imaging (MRI) reveal three major patterns of disease. Gallbladder carcinoma could present as a mass that completely replaces the gallbladder and invades the adjacent liver or as an intraluminal enhancement mass (in 25% of cases) arising from the fundus (60%) or body (30%) [15,16,17,18]. Regarding our six patients, two of them presented with a mass that replaced a part of gallbladder and invaded the adjacent liver (Case 3 and 6) and one of them as a suspicious intraluminal gallbladder lesion localized in gallbladder fundus on CT and MRI (Case 5).

A third presentation of gallbladder carcinoma is either irregular focal or diffuse wall-thickening of the gallbladder [15,19]. Regarding our cases, three of them had presented with this imaging scenario (Cases 1, 2 and 4).

Tumor can spread to the liver (65%), colon (15%), duodenum (15%) and pancreas (6%) [12,15]. Regarding our cases, in Case 1 the tumor spread to the liver and duodenum and transvers colon and in Cases 4 and 6 metastatic spread to the liver and duodenum can be noted.

Tumor extending to biliary tract is associated with poor prognosis. This aspect was presented in Case 3. Also, in Cases 1, 3 and 6, local lymphatic tumoral spread was presented [15,20].

Associated findings include a checklist of:

✓ gallstones (Cases 1, 2);
✓ biliary dilatation (Case 3);
✓ metastases in the liver parenchyma (segments IV, V) (Cases 1, 3);
✓ peritoneum;
✓ bulky porta hepatis, adenopathy (Cases 1, 3, 5);
✓ invasion of the liver and bowel (Cases 1, 3, 4, 5, 6) [15,18].

Adenocarcinoma is the most common morphologic subtype of gallbladder cancer (over 90% of cases), followed by adenosquamous and squamous cell type (10–15%). Small cell carcinoma, neuroendocrine cell tumors and metastases are the rare types [10]. In our six cases, different subtypes of gallbladder cancer were observed, three of which were adenocarcinoma (Cases 1, 4 and 6). In two cases (Cases 3 and 5), the histopathology showed small cell neuroendocrine carcinoma. Neuroendocrine carcinoma of gallbladder is a rare entity and it tends to be more aggressive compared with gallbladder adenocarcinoma [21].

In our Case 5 report (Figure 7), the patient presented gallbladder carcinoma with endocrine manifestation. Gallbladder cancer associated with SIADH syndrome represents a very rare entity with few cases reported in the current literature. Hyponatremia (<135 mmol/L) is correlated with a negative prognosis and in some case is a predictive factor for cancer patients. Paraneoplastic syndrome of inappropriate antidiuretic hormone secretion (SIADH) is induced by the abnormal secretion of antidiuretic hormone by tumoral cells [21]. In our case, the final diagnosis was gallbladder carcinoma associated with SIADH as a paraneoplastic syndrome.

Moreover, the American Joint Committee on Cancer 8th edition gallbladder cancer staging system is staged by the depth of tumor invasion (T), presence of lymph node metastases (N) and presence of distant metastases (M) [1]. The T component describes the depth that the tumor has grown from the inside through the outer layers. The N component indicates invasion in lymph nodes. The M component describes distant metastases, the most common sites of metastases being represented by the peritoneum and liver parenchyma [10,22].

Furthermore, for the most important imaging part regarding differential diagnostic, imaging represents a helpful modality for distinguishing between benign and malign gallbladder diseases, in most cases [15,23]. Differential diagnosis includes a group of diseases, such as complicated or chronic cholecystitis, xanthogranulomatous cholecystitis, adenomyomatosis, adenoma, porcelain and metastases [15].

A gallbladder tumor is usually represented on imaging as focal or diffuse asymmetric mural thickening [10,24].

The presence of symmetric wall thickening often indicates a benign origin, such as acute or chronic cholecystitis or adenomyomatosis [24].

Acute cholecystitis complicated by pericholecystic abscess are frequently differentiated from gallbladder cancer due to their typically rapid and severe acute clinical presentation. Also, acute cholecystitis on contrast-enhanced CT shows increased gallbladder wall enhancement associated with hyperemia, frequently associated with gallstones (Figure 11) [10].

Moreover, the differential diagnosis between xanthogranulomatous cholecystitis (Figure 12) and gallbladder tumor, can be challenging, particularly in patients with proliferative fibrosis. Xanthogranulomatous cholecystitis is an uncommon form of chronic cholecystitis characterized by aggressive inflammatory changes, by intramural hypoattenuating nodules and by fat detection on MRI in thickened wall (Figure 13) [25,26,27,28,29].

Furthermore, gallbladder adenomyomatosis is a benign gallbladder lesion. Imaging shows a focal or diffuse gallbladder mural thickening, which can mimic cancer. The invaginations or diverticula are frequently called Rokitansky–Aschoff sinuses, which can be easily visualized on MRI imaging [19,20,30]. The differential diagnosis for intraluminal polypoid tumors includes both benign and malignant lesions: adenomatous polyp, cholesterol polyp, carcinoid tumor and metastasis from melanoma or renal cell carcinoma [31].

Metastases to the gallbladder are rare, usually with a late diagnosis and represent an end-stage of malignancy, being commonly associated with metastases to other tissues (patients with an established diagnosis of disseminated cancer) and usually presenting poor and unfavorable prognosis. The most common primary tumor metastasizing to the gallbladder is melanoma (55% of cases) (Figure 14, Figure 15 and Figure 16), followed by breast cancer (13%), hepatocellular carcinoma (13%) and renal cell carcinoma (7%) [32,33].

## Figures and Tables

**Figure 1:**
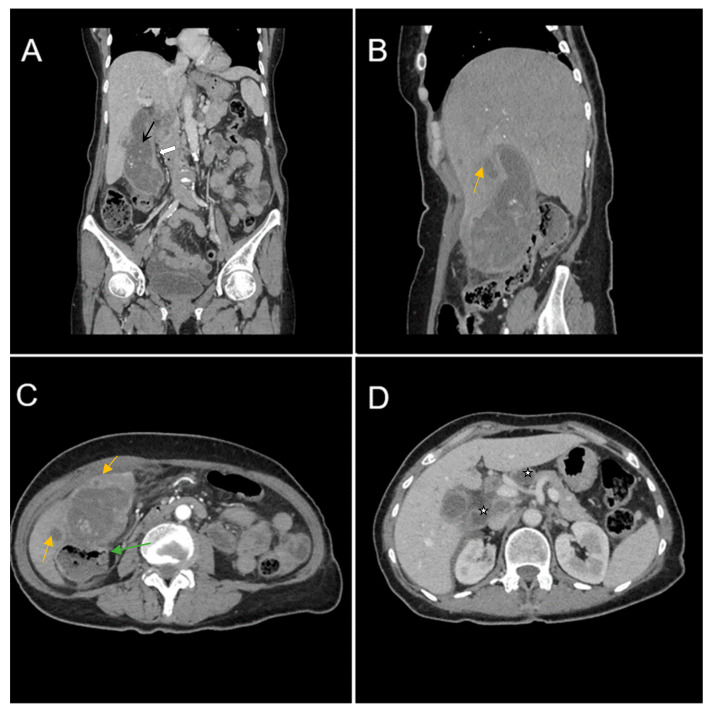
Multiplanar sections of contrast-enhanced CT acquisitions richly illustrating a low differentiated gallbladder adenocarcinoma. (**A**) Gallbladder hydrops (>40 mm transverse measurement, 142 mm longitudinal measurement) with asymmetric gallbladder mural thickening, 7 mm (white arrow), and multiple intraluminal mixed stones, 5–8 mm (black arrow). (**B**,**C**) Liver metastases—hypodense nodular hepatic lesions with rim contrast enhancement (yellow arrow). (**C**) Tumoral extension into IV, V segments of the right hepatic lobe and contact with the ascending colon (green arrow). (**D**) Lymphatic metastases (white stars).

**Figure 2:**
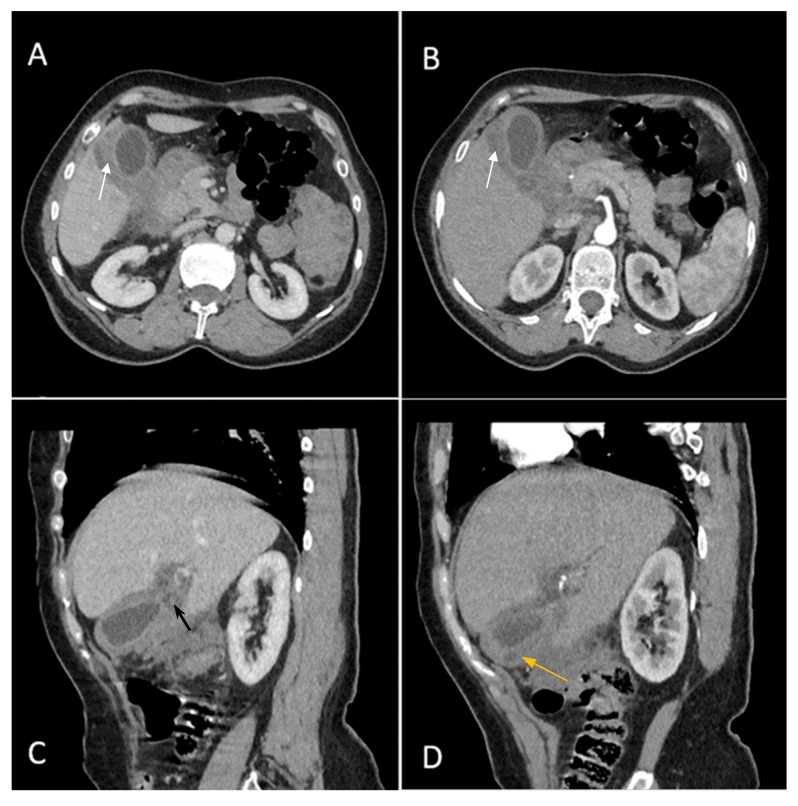
Multiplanar sections of contrast-enhanced CT acquisitions richly illustrating gallbladder carcinoma. (**A**,**B**) Gallbladder with irregular mural thickening, 16 mm (white arrow), pericholecystic fluid and loco-regional inflammatory lymph nodes. (**C**) Gallbladder with an intraluminal gallstone, 15 mm (black arrow). (**D**) Abscess adjacent to the gallbladder with subtle peripheral contrast enhancement (yellow arrow).

**Figure 3:**
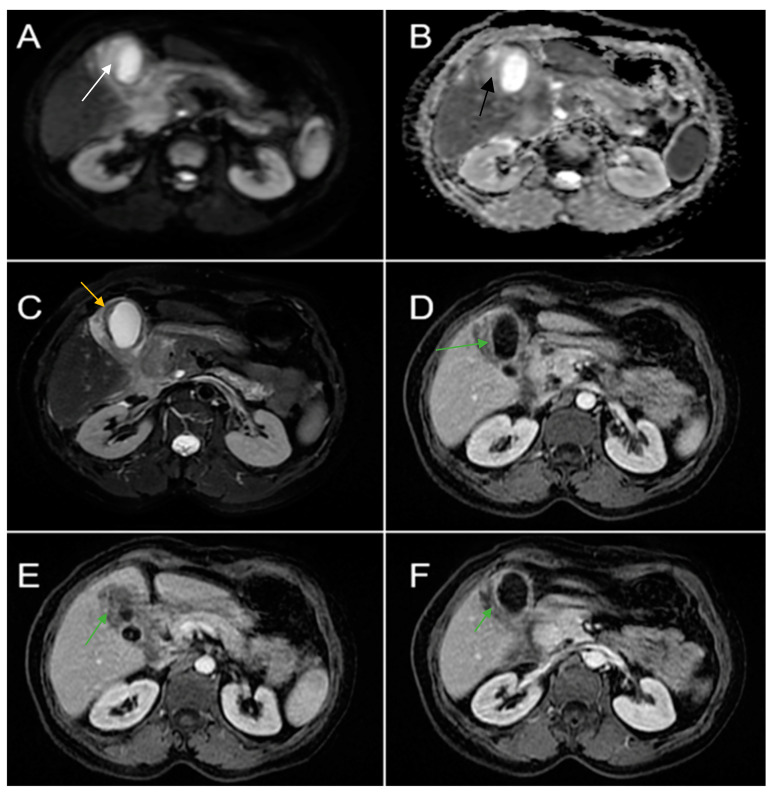
Abdominal MRI sequences highlighting imaging features suggestive of gallbladder carcinoma. (**A**) Diffusion-weighted imaging (DWI B800) showing bright high signal intensity of the wall thickening of the gallbladder (white arrow). (**B**) On apparent diffusion coefficient (ADC) map, the wall thickening is dark (black arrow)—illustrating markedly diffusion restriction—which in correlation with increased levels of CEA and CA 19-9 is highly suggestive of gallbladder carcinoma. (**C**) Axial T2-weighted FIESTA showing asymmetric strongly inhomogeneous wall thickening involving the gallbladder (yellow arrow). (**D**–**F**). Axial contrast-enhanced T1-weighted images showing heterogeneous enhancement of the wall thickening (green arrows).

**Figure 4:**
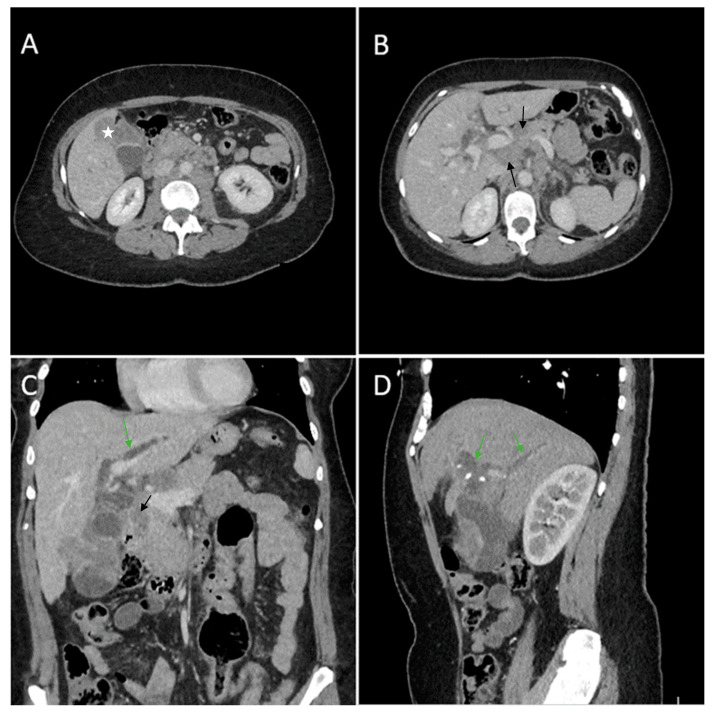
Multiplanar sections of contrast-enhanced CT acquisitions richly illustrating gallbladder carcinoma. (**A**) Heterogeneous ill-defined intraluminal irregular mass located predominantly in the gallbladder fundus (white star). (**B**) Multiple lymphadenopathies with areas of necrosis included (black arrows). (**C**) Gallbladder mass presents extension in the surrounding liver (segment V). (**D**) Intrahepatic biliary dilatation in both hepatic lobes, predominantly perihilar (green arrows).

**Figure 5:**
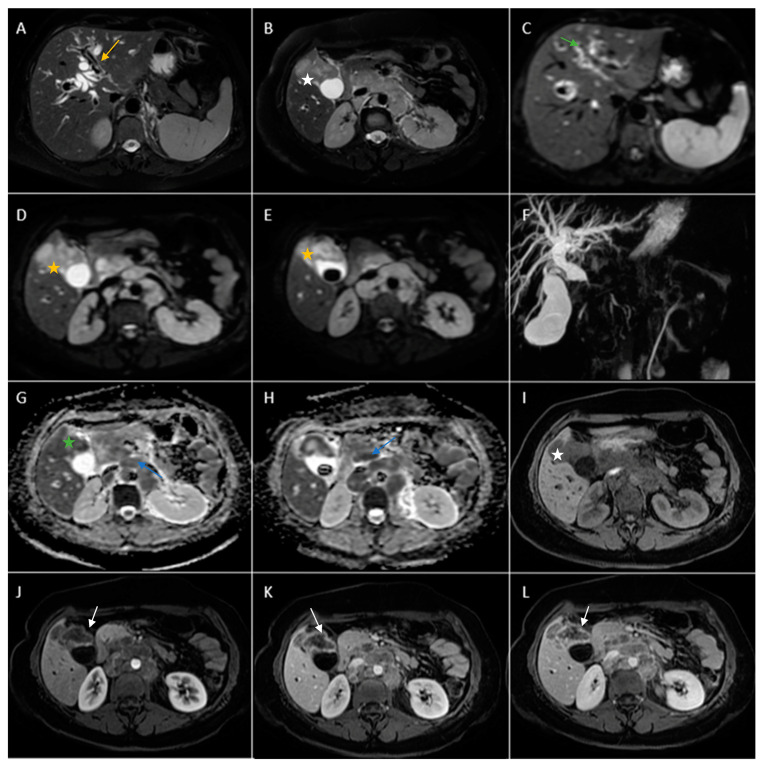
Abdominal MRI vividly illustrating synchronous gallbladder and biliary tract carcinoma with multiple large lymphadenopathies localized in the hepatic hilum, surrounding the cephalic region of the pancreas and in the celiac region. (**A**) Axial T2-weighted FS showed significant intrahepatic biliary dilatation in both hepatic lobes (yellow arrow). (**B**) Axial T2-weighted FS showed hypointense intraluminal gallbladder mass (white star) and multiple large lymphadenopathies. (**C**) Axial diffusion-weighted imaging (DWI B800) showed irregular, asymmetrical thickening of the walls of the intrahepatic bile ducts with high signal intensity suggestive of cholangitis (green arrow). (**D**,**E**). DWI B800 highlighted the gallbladder mass; inhomogeneous areas of high signal (yellow stars). (**F**) Coronal 3D MRCP showed enlarged gallbladder with an intraluminal gallstone and dilated intrahepatic and extrahepatic biliary tree. (**G**,**H**). On apparent diffusion coefficient (ADC) map, the gallbladder mass is dark, illustrating markedly diffusion restriction (green star). Multiple large lymphadenopathies are also observed mainly in the lombo-aortic region, in the cephalic pancreatic region and in the hepatic hilum (blue arrow). (**I**) Axial T1-weighted image showing hypointense irregular tumoral gallbladder mass (white star). (**J**–**L**). Axial contrast-enhanced (arterial phase followed by venous phase) T1-weighted image showing rim-enhancing of the tumoral gallbladder mass (white arrow).

**Figure 6:**
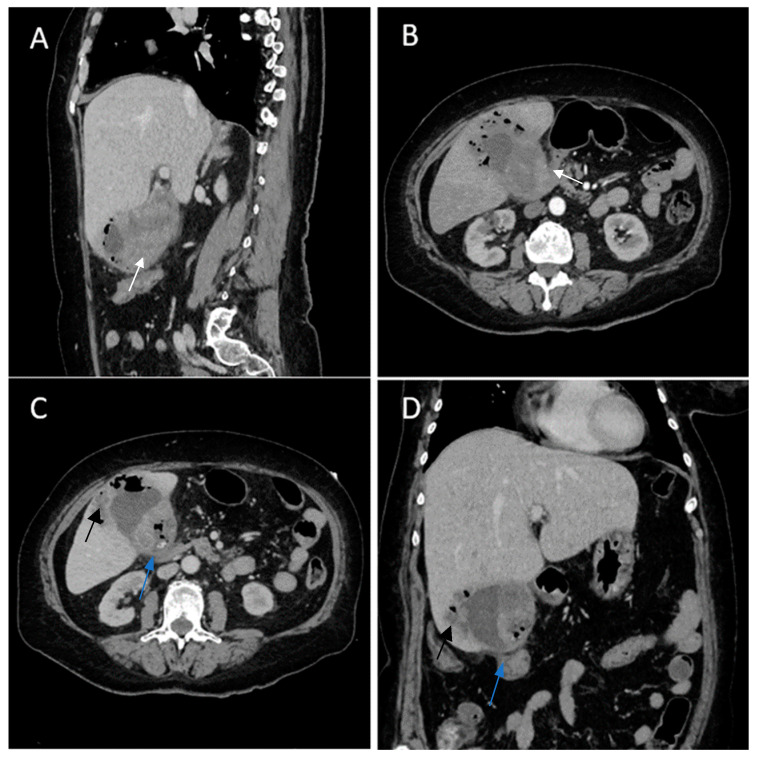
Multiplanar sections of contrast-enhanced CT acquisitions richly illustrating gallbladder carcinoma with an associated necrotizing infectious component. (**A**,**B**). Distended gallbladder with asymmetrical thick-walled gallbladder (16 mm) (white arrow). (**C**,**D**). Abscess adjacent to the gallbladder (black arrow); extension to the duodenum (blue arrow).

**Figure 7:**
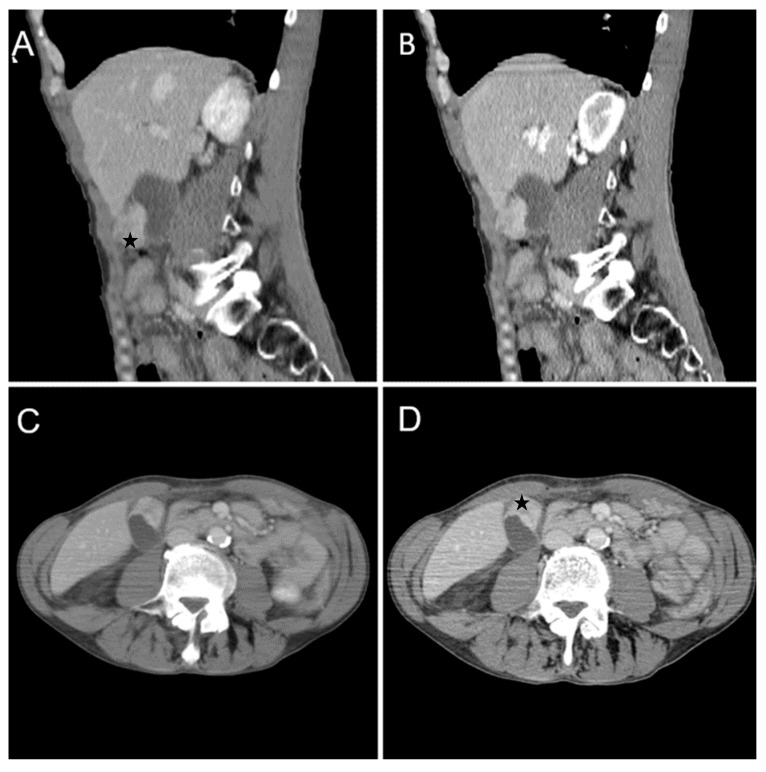
Multiplanar sections of contrast-enhanced CT acquisitions richly illustrating gallbladder carcinoma. (**A**–**D**). Heterogeneous, contrast-enhancing intraluminal gallbladder mass located in the gallbladder fundus region (black star).

**Figure 8:**
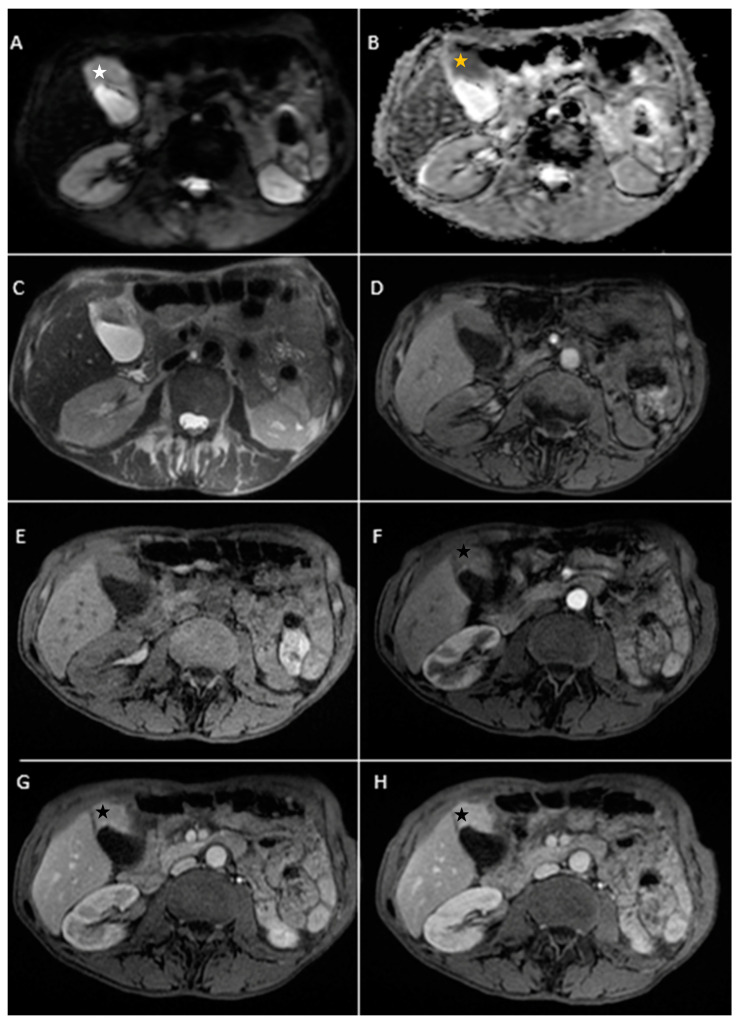
Abdominal MRI sequences highlighting gallbladder carcinoma. (**A**) Diffusion-weighted imaging (DWI B800) showing areas of moderate-high signal of the intraluminal gallbladder mass located in the fundus area (white star). (**B**) On ADC map, the intraluminal gallbladder mass is dark—diffusion restriction (yellow star). (**C**,**D**). Axial T2-weighted showing distended gallbladder with a heterogeneous hypointense intraluminal mass and axial T1 dual ECHO showing isointense gallbladder mass. (**E**) Axial T1-weighted image showing isointense gallbladder mass. (**F**–**H**). Axial contrast-enhanced T1-weighted image (arterial phase followed by venous phase) showing strong contrast-enhancement of the intraluminal gallbladder mass (black star).

**Figure 9:**
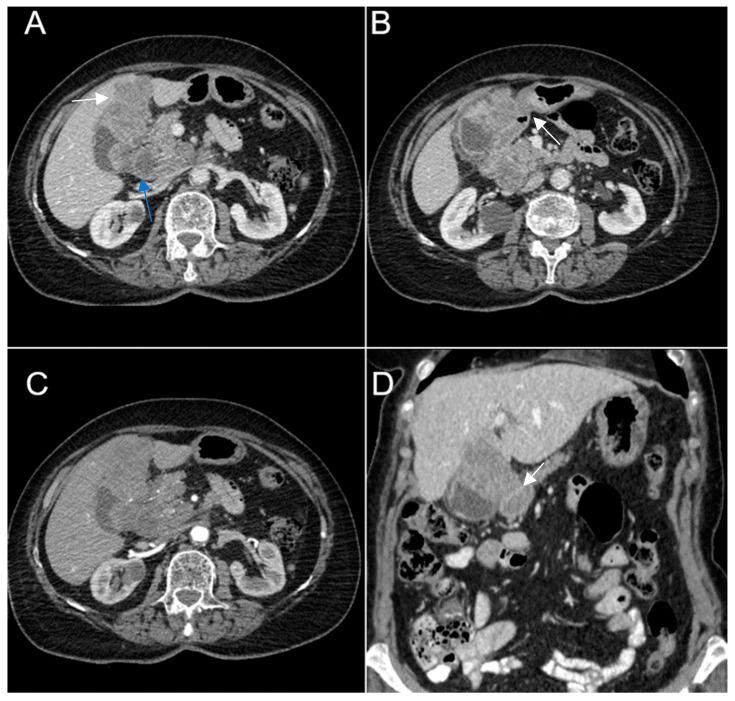
Multiplanar sections of contrast-enhanced CT acquisitions richly illustrating gallbladder carcinoma. (**A**–**D**). A large, inhomogeneous pseudonodular mass with heterogeneous contrast enhancement, with invasion in the adjacent liver, pyloric antrum and duodenum II (white arrows). (**A**) Lymphatic metastases; with compressive effect on the inferior vena cava and right renal artery and vein (blue arrow).

**Figure 10:**
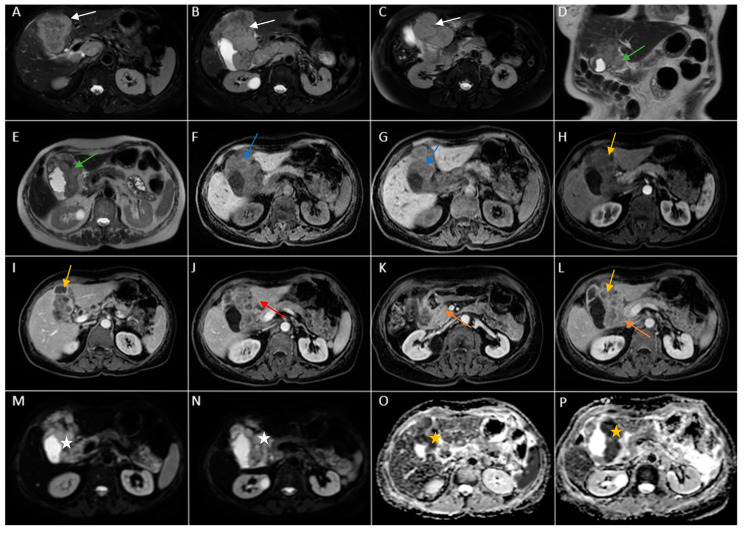
Abdominal MRI images vividly illustrating epithelial gallbladder carcinoma. (**A**–**C**). Axial T2-weighted FS showing inhomogeneous moderate hypointense heterogeneous tumoral parietal mass surrounding the gallbladder (white arrow). (**D**,**E**). Coronal and axial T2-weighted images showing hypointense heterogeneous tumoral gallbladder mass (green arrow). (**F**,**G**). Axial T1-weighted illustrating iso-hypointense tumoral mass (blue arrow). (**H**–**L**). Axial contrast-enhanced T1-weighted image (arterial phase followed by venous phase) showing a heterogeneous enhancement of the gallbladder mass with areas of necrosis (yellow arrows). The mass invades the adjacent liver ((**J**), red arrow) and duodenum II ((**K**,**L**), orange arrows). (**M**,**N**). Diffusion-weighted imaging (DWI B800) showing bright signal of the large gallbladder mass (white stars). (**O**,**P**). On ADC map, the large gallbladder mass is dark, illustrating diffusion restriction (yellow stars).

**Figure 11:**
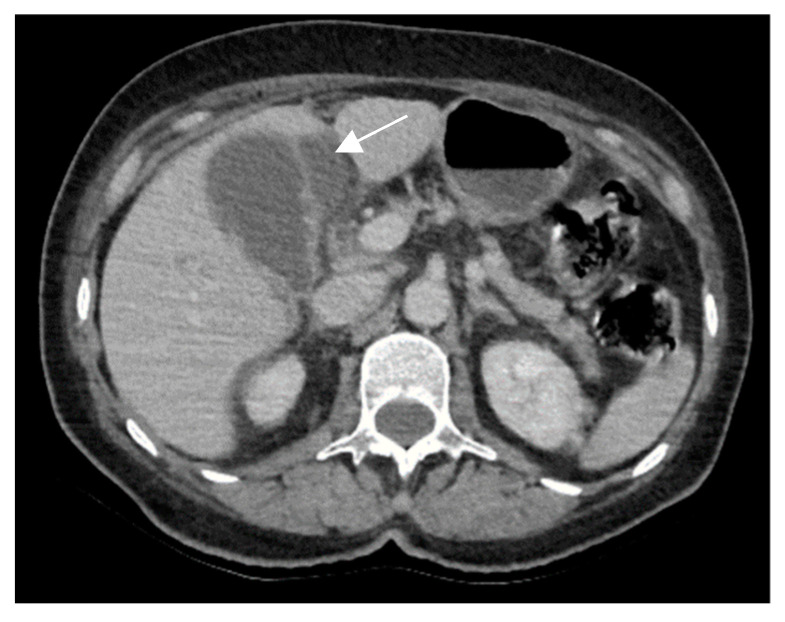
A 67-year-old woman undergoing contrast-enhanced CT for suspected abdominal acute appendicitis. CT images show pericholecystic abscess (white arrow) and symmetric wall thickening suggestive of acalculous cholecystitis.

**Figure 12:**
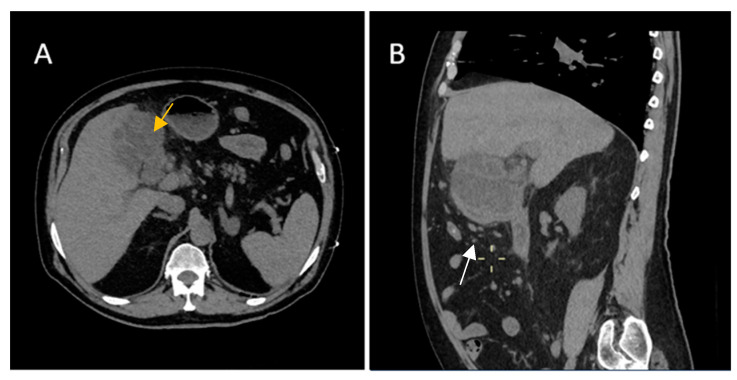
A 70-year-old male with a history of hypertension, type 2 diabetes, peripheral venous thrombosis, presented at our Emergency Department with abdominal pain accompanied by diarrhea, night sweats and fever. His laboratory findings showed elevated inflammatory markers. Computerized tomography (CT) revealed (**B**)—diffuse wall thickening with intramural low-density nodules and bands in thickened walls (white arrow) associated with (**A**) pericholecystic inflammatory change (yellow arrow).

**Figure 13:**
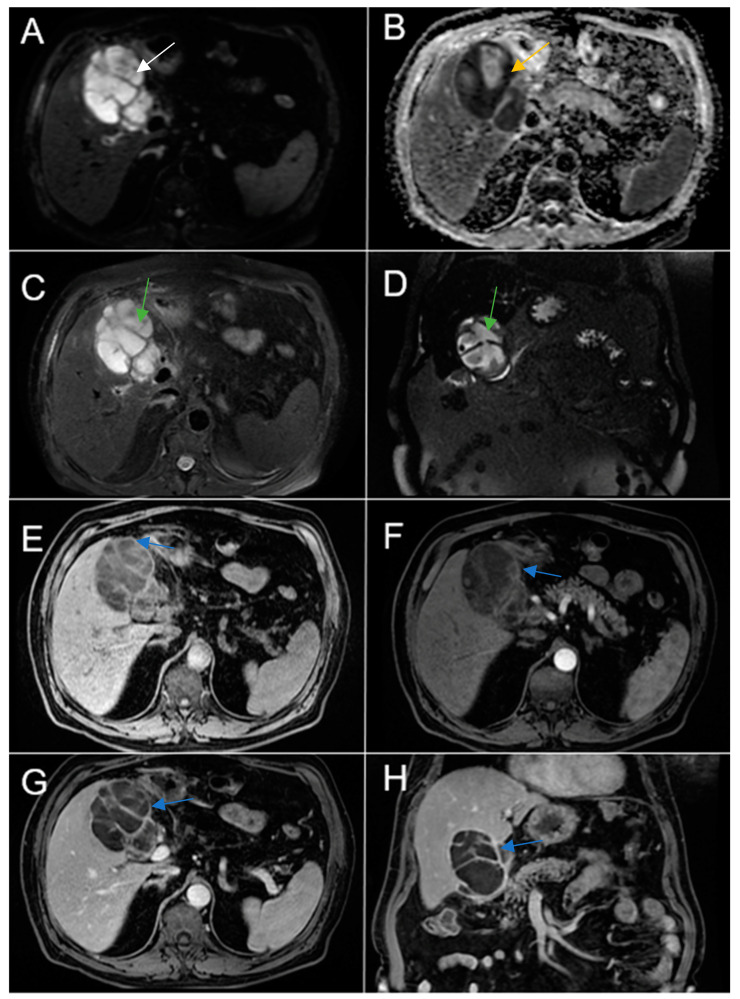
Abdominal MRI vividly illustrating Xanthogranulomatous Cholecystitis. (**A**) Diffusion-weighted imaging (DWI B800—bright high signal) demonstrated restricted diffusion (white arrow), but malignancy typically demonstrates lower ADC values. (**B**) On ADC map, the wall thickening is dark (yellow arrow). (**C**,**D**). Intramural areas of necrosis are high signal intensity on axial and coronal T2-weighted images (green arrows). (**E**–**H**). Axial unenhanced and contrast-enhanced T1-weighted images showing diffusely thickened wall, with multiple intramural nodules with peripheral contrast enhancement (blue arrows).

**Figure 14:**
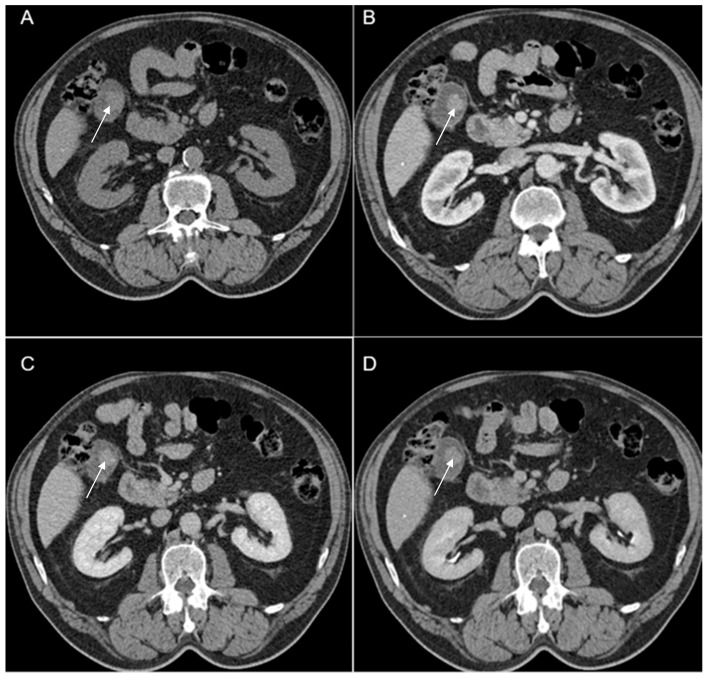
We present a rare and unusual case of a 67-year-old male with a medical history of cutaneous melanoma on right thorax stage IV. CT images fully illustrated a contrast-enhancing polypoid pseudonodular mass located in the gallbladder fundus measuring 21/20 mm (white arrows). (**A**) Native examination: 55–60 HU. (**B**) Arterial phase: 95–119 HU. (**C**) Venous phase: 80–100 HU. (**D**) Delayed phase (3 min): 75–80 HU. MRI was performed (Figure 15). Melanin is usually hyperdense on unenhanced CT images and hyperintense on T1-weighted MRI; this criterion plays an essential role in the differential diagnosis between primary or secondary gallbladder lesions. The patient followed immunotherapy.

**Figure 15:**
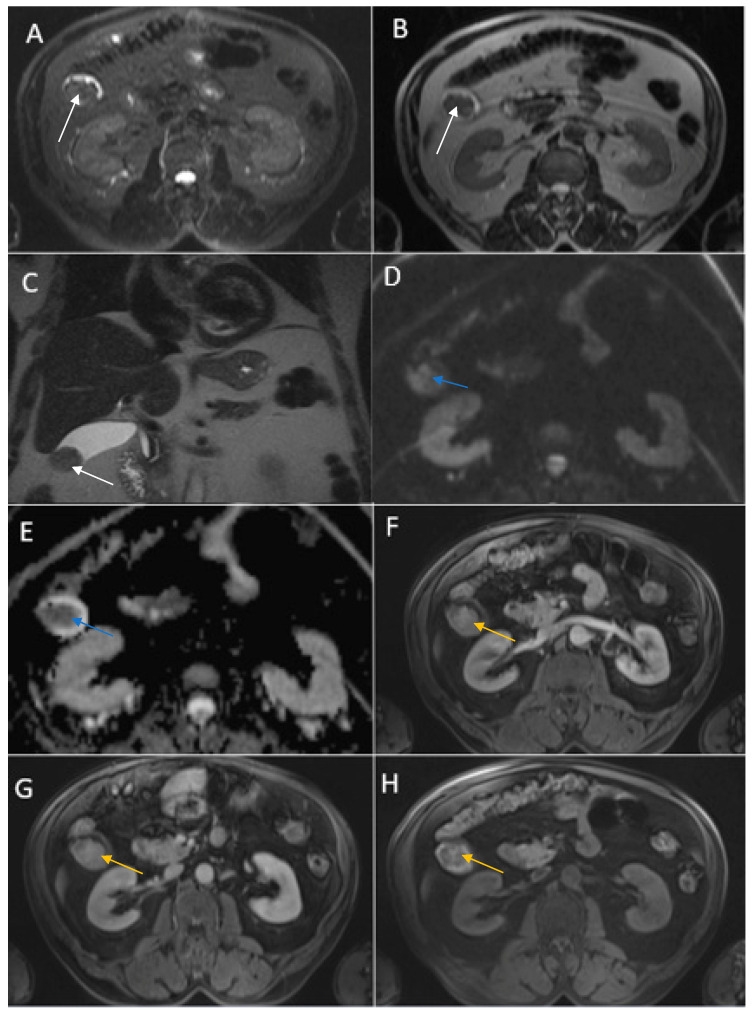
Abdominal MRI. (**A**) Axial T2-weighted FS showing markedly hypointense pseudonodular tumoral gallbladder lesion (white arrow). (**B**) Axial T2-weighted showing hypointense gallbladder lesion (white arrow). (**C**) Coronal T2-HASTE hypointense gallbladder lesion (white arrow). (**D**) Diffusion-weighted imaging (DWI B800) showing bright hypersignal of the lesion (blue arrow). (**E**) Restricted diffusion (blue arrow) on apparent diffusion coefficient (ADC) map. (**F**,**G**) Axial contrast-enhanced T1-weighted image showing a strongly enhancing polypoid pseudonodular mass in the gallbladder fundus. (**H**) Axial T1-weighted image FS showing hyperintense nodular lesion component (yellow arrows), highly suggestive of melanin.

**Figure 16:**
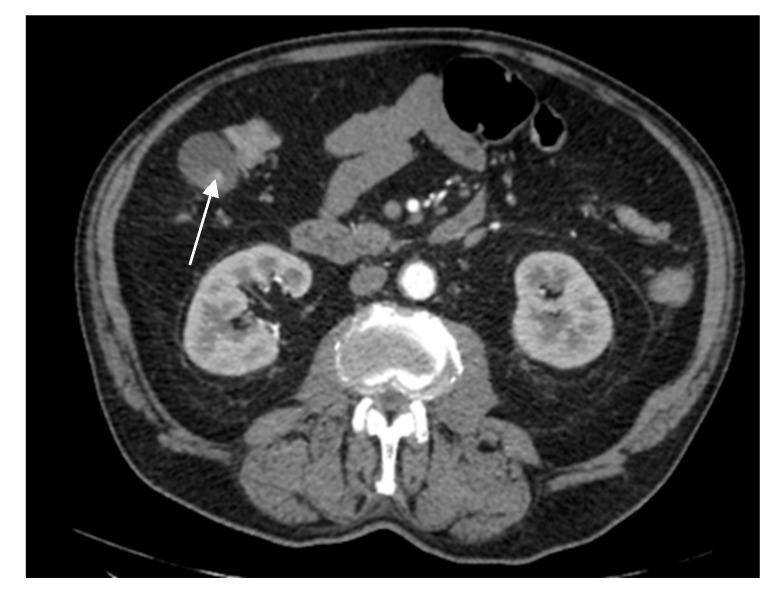
Remarkable treatment response to immunotherapy; intracholecystic metastatic lesion from cutaneous melanoma after immunotherapy appears in remission (white arrow).

## Data Availability

The data presented in this study are available on request from the corresponding author (N, L.C.).
